# Long-term outcomes of small head metal-on-metal compared to ceramic-on-polyethylene primary total hip arthroplasty: a registry-based cohort study

**DOI:** 10.1007/s00264-025-06437-z

**Published:** 2025-02-12

**Authors:** Amanda I. Gonzalez, Christophe Barea, Matthieu Zingg, Guido Garavaglia, Robin Peter, Pierre Hoffmeyer, Didier Hannouche, Anne Lübbeke

**Affiliations:** 1 Division of Orthopaedics and Trauma Surgery, Geneva University Hospitals, Geneva, Switzerland; 2 SWISS Foundation for Innovation and Training in Surgery, Geneva, Switzerland; 3 Nuffield Department of Orthopaedics, Rheumatology and Musculoskeletal Sciences, University of Oxford, Oxford, UK

**Keywords:** Total hip arthroplasty, Metal-on-metal, Ceramic-on-polyethylene, Adverse local tissue reaction

## Abstract

**Purpose:**

We aimed to compare the long-term outcomes of small-head (28 mm) metal-on-metal (MoM) total hip arthroplasty (THA) to ceramic-on-polyethylene (CoP) THA using the same cup.

**Methods:**

All primary elective MoM and CoP THAs performed 1998–2011 were prospectively included in a local registry. Patients were followed until 31 December 2022. Outcomes were all-cause revision, complications and mortality. The uncemented Morscher 28 mm monobloc press-fit cup was used in all THAs.

**Results:**

Overall, 3257 THAs were included, 864 MoM (mean age 63) and 2393 CoP THAs (mean age 72). Mean follow-up of the cohort was 12.9 years (maximum 26.8 years). Revision for any cause was performed in 85 MoM and 79 CoP THAs. Cumulative incidence of all-cause revision at 20 years was 13.2% (95% CI 10.6 to 16.3) in MoM and 6.3% (95% CI 4.8 to 8.3) in CoP group. Adjusted hazard ratio for all-cause revision was 1.88 (95% CI 1.34 to 2.65) comparing MoM vs. CoP. Diagnoses at revision were mainly aseptic loosening (33%) and adverse local tissue reactions (33%) in MoM and aseptic loosening in CoP group (44%). The smoothed hazard function revealed the largest difference in instantaneous revision rate between three and 14 years postoperative. After that period no difference was observed.

**Conclusion:**

Overall, the cumulative risk of all-cause revision was almost twice as high in patients with a small head MoM as compared to a CoP THA over the 20-year period. However, most of the excess in revisions among MoM patients occurred between three and 14 years postoperative.

## Introduction

High mid-term revision rates and adverse local tissues reaction (ALTR) following metal-on-metal (MoM) total hip arthroplasty (THA) were mostly reported with large head MoM THAs [1, 2]. A few studies reported increased revision rates and ALTR with small MoM THAs [3–8]. Reported revision rates for small head MoM THAs were less than 5% at five years but greater than 11% at 20 years [9]. The sample sizes of the studies reporting on outcomes of small head MoM THAs with more than tenyear follow up were mostly small [3, 10–15] with numbers of THAs included ranging from 52 to 180. Furthermore, comparative studies (e.g. MoM vs. other bearing surface) are sparse, and mostly limited to a follow-up less than ten years [16–18].

Although there is a decrease in the use of MoM bearings [19, 20], still a high number of patients have a THA with a MoM bearing implanted, leading to the publication of guidelines for surveillance of MoM THAs [21], which included clinical, laboratory and imaging studies [22]. Also, questions and concerns remain concerning the long-term outcome (including death) and need for surveillance of patients with small head MoM THA.

Our objective was to compare the occurrence of long-term complications (all-cause revision, any reoperation, infection, dislocation and periprosthetic fracture) and mortality after small-head (28 mm) MoM THA vs. ceramic-on-polyethylene (CoP) THA with the same cup, an uncemented Morscher monobloc 28 mm press-fit cup.

## Materials and methods

### Study design and study population

We included all primary small head MoM and CoP THAs performed between 03/1998 and 06/2011 at a large University Hospital. Since March 1996 all patients undergoing a THA are prospectively enrolled in the hospital-based arthroplasty registry. Completeness of recording of THAs is > 99% based on validation against the hospital’s diagnosis coding system, completeness of revision capture is > 96% based on data from the Swiss national joint registry (SIRIS). In a previously published article [23], we evaluated the results up to 12 years after surgery for these prostheses, end of follow-up being 31 December 2012. We updated the outcomes of this cohort with a follow-up until 31 December 2022. THAs performed for fracture or metastatic disease were excluded. Overall, 2785 patients met the inclusion criteria, after exclusion of 47 patients who had refused to consent, 3257 primary THAs in 2738 patients were included, 864 MoM and 2393 CoP THAs. The hospital-based arthroplasty registry was approved by the ethics committee of our institution (reference no. CER: 05–017 (05-0419)).

### Exposure

All THAs received the same cup, a Morscher press-fit, uncemented, and monoblock acetabular cup (Zimmer Ltd, Winterthur, Switzerland). According to surgeon’s preference either a metal-on-metal bearing or ceramic-on-polyethylene bearing was used. Both types of bearings were available during the inclusion period. The implants were described as follows in our previously published paper [23]. The uncemented monobloc Morscher press-fit acetabular component (Zimmer Ltd, Winterthur, Switzerland) was used for all patients. This has a flexible titanium mesh shell backing which is bonded directly to an outer ultrahigh-molecular-weight polyethylene (UHMWPE) surface to eliminate the potential for backside wear. Two types of bearing were used according to surgeon preference. One was a metal-on-metal (MoM) bearing (Metasul, Zimmer Ltd) composed of a high-carbon (N 0.2%) cobalt–chromium alloy 28 mm femoral head which articulates with a high-carbon (N 0.2%) cobalt-chromium alloy inlay embedded into the UHMWPE liner. The second choice was a conventional UHMWPE bonded directly to the titanium mesh shell, combined with a third generation alumina ceramic 28 mm (Sulox, Zimmer Ltd) diameter head (CoP bearing). The acetabular components were sterilized by gamma irradiation in an inert atmosphere. The cemented stems included the Müller straight stem and the Virtec stem, both manufactured from Protasul-10 alloy (CoNiCrMo) (both Zimmer). The uncemented stems were the Spotorno CLS, Protasul-100 titanium alloy or the Wagner Conus, Protasul-64 titanium alloy (both Zimmer). For cemented stems, gentamicin-loaded bone cement was used, and cementing was performed using a third-generation technique.

### Outcomes

The following outcomes were assessed: all-cause revision, presence of ALTR at revision, any other reoperation, prosthetic joint infection, dislocation, periprosthetic fracture, and mortality. Revision surgery was defined as any change to a component of the arthroplasty (cup, head, and/or stem), while reoperation included any type of surgery not involving a change of component of the arthroplasty (for instance: arthroplasty irrigation/debridement, osteosynthesis, abductor reinsertion). ALTR was defined based on the perioperative findings and pathology report. Periprosthetic fracture included any fracture diagnosed postoperatively. Dislocation included any diagnosis for dislocation.

### Covariates

The following covariates were evaluated: age, sex, preoperative Body Mass Index (BMI), American Society Anaesthesiology (ASA) score, comorbidity count (beside osteoarthritis), diabetes (present or absent), smoking status (never vs. ever-smoker), diagnosis (primary vs. secondary osteoarthritis), stem fixation (cemented vs. uncemented), and Charnley score. Comorbidity count was classified in less than four or ≥ 4 [24]. Ever smokers included former and current smokers.

### Data collection

Patients included in the local hospital-based arthroplasty registry have their information on preoperative status and surgical intervention routinely documented by the operating surgeon on specific designed data collection forms. Information about co-morbidities is retrieved from anesthesia records and discharge summaries by a medical secretary. Outcomes (all-cause revision, any other reoperation, PJI, dislocation and periprosthetic fracture) are collected by the operating surgeon or during the telephone interview if performed outside of our hospital [25].

### Statistics

Continuous variables were assessed for normality and reported as mean with standard deviation and compared with the t-test. Categorical variables were reported as proportions. To compare the two bearing surface groups Pearson Chi-Square tests was used.

To compare the outcomes, incidences rates were calculated and expressed in person-years. The person-time for the outcomes was the length between date of surgery and the outcome, revision for any reason, loss of follow up, death, or end of follow up (December 31, 2022) whatever came first. Unadjusted and adjusted hazard ratios (HR) with 95% confidence intervals (CI) were calculated with a Cox regression model. Adjustment was performed for sex, age at surgery, diagnosis, type of stem fixation, number of diseases, BMI, ASA score and smoking status.

The cumulative risks of all-cause revision (CRR) were visualized by bearing surface using Kaplan-Meier analysis. Smoothed hazard estimates were obtained with their 95% CIs to evaluate whether the timing of the revisions differed between the two groups [26]. Hazard estimates quantify the immediate risk, in this case of all-cause revision, attached to an individual known to be alive at time t. All analysis were performed using a statistical package PASW statistics version 25 (Chicago: SPSS Inc.) and Stata version 17 (Stata Corp., College Station, Texas).

## Results

The two groups differed at baseline concerning sex, age, ASA score, number of diseases, smoking status, type of osteoarthritis, and stem fixation. Patients in the MoM group were predominantly males, younger, in better health, smoked more often, had more secondary OA and an uncemented stem (Table 1). Mean follow up time was 13.5 (SD 5.6, maximum 24.1) years in the MoM group and 12.7 (SD 6.5, maximum 26.8) years in the CoP group.

**Table 1:** Baseline characteristics

	MoM THA *N* = 864 (26.5%)	CoP THA *N* = 2393 (73.5%)	*p*
Woman (%)	382 (44.2)	1411 (59)	< 0.001
Age, mean (SD)	63.2 (11.1)	72.0 (10.0)	< 0.001
Age (%)			
< 55 yrs	164 (19.0)	124 (5.2)	
55 to 74.9 yrs	567 (65.6)	1222 (51.1)	
≥ 75 yrs	133 (15.4)	1047 (43.8)	< 0.001
BMI, mean (SD)	27.3 (4.9)	27.0 (4.5)	0.078
BMI ≥ 30 (%)*	232 (26.9)	571 (24.2)	0.118
ASA**			
1	147 (17.0)	191 (8.0)	
2	586 (67.8)	1506 (63.0)	
3–4	131 (15.2)	695 (29.1)	< 0.001
Comorbidity count (%)			
0–3	809 (93.6)	2144 (89.6)	
≥ 4	55 (6.4)	249 (10.4)	< 0.001
Diabetes, yes (%)	71 (8.2)	264 (11.0)	0.0192
Smoking never*** (%)	440 (53.8)	1345 (62.7)	< 0.001
Primary OA (%)	661 (76.5)	2039 (85.2)	< 0.001
Stem fixation cemented (%)	668 (77.3)	2296 (95.9)	< 0.001
Charnley****			
A	319 (37.2)	800 (33.6)	
B	316 (36.8)	8628 (36.2)	
C	223 (26.0)	716(30.1)	0.016

* Missing information on BMI for 2 patients in the MoM group and 32 patients in the CoP group

** Missing information on ASA score for one patient in the CoP group

***Missing information on smoking status for 46 patients in the MoM group and 247 patients in the CoP group

****Missing information on Charnley status for 6 patients in the MoM group and 15 patients in the CoP group

Revision for any cause was performed in 85 MoM at a mean time of 100.7 months (SD 55.2) and in 79 CoP THAs at a mean time of 112.8 months (SD 86.7). The adjusted HR for all-cause revision was 1.9 (95% CI 1.3 to 2.7) (Table 2). Diagnosis at revision differed particularly regarding the presence of ALTR. ALTR was diagnosed in 36 patients with MoM bearing (Table 3). Diagnosis dislocation as cause of the revision differed also between the two groups (Table 3), median time for revision with diagnosis dislocation was 94 months (IQR 14–147) for the CoP group and 57 months (IQR 1-141) for the MoM group. After stratification by sex adjusted HR for all-cause revision was 2.1 (95% CI 1.2 to 3.7) for women and 1.7 (95% CI 1.1 to 2.6) for men.

**Table 2:** Outcomes

	Metal-on-metal *N* = 864	Ceramic-on-polyethylene *N* = 2393	Unadjusted HR (95% CI)	Adjusted HR (95% CI)*
All-cause revision				
Cases/1000 person-years	85/11652.6 × 10^3^	79/30323.8 × 10^3^		
Incidence rate	7.29	2.61	2.81 (2.07 to 3.83)	1.88 (1.34 to 2.65)
Any other reoperation				
Cases/1000 person-years	30/12036.0 × 10^3^	66/30339.5 × 10^3^		
Incidence rate	2.49	2.17	1.21 (0.78 to 1.86)	1.12 (0.68 to 1.82)
Prosthetic joint infection				
Cases/1000 person-years	17/12126.7 × 10^3^	21/30797.2 × 10^3^		
Incidence rate	1.40	0.68	2.23 (1.17 to 4.24)	2.05 (1.01 to 4.14)
Dislocation				
Cases/1000 person-years	29/12014.0 × 10^3^	69/30231.7 × 10^3^		
Incidence rate	2.41	2.28	1.13 (0.73 to 1.75)	0.99 (0.60 to 1.63)
Periprosthetic fracture				
Cases/1000 person-years	16/11937.3 × 10^3^	34/30110.4 × 10^3^		
Incidence rate	1.34	1.13	1.22 (0.671 to 2.22)	1.04 (0.52 to 2.09)

*Adjustment performed for sex, age at surgery, diagnosis, type of stem fixation, comorbidity count, BMI, ASA score and smoking status

**Table 3:** Diagnosis for all-cause revision

	MoM THA *N* = 108 (%)	CoP THA *N* = 81 (%)
Aseptic loosening of any component	36 (33.3)	36 (44.4)
Cup	3	15
Stem	9	2
Stem and cup	24	19
Adverse local tissue reaction	36 (33.3)	0
Prosthetic joint infection	13 (12.0)	15 (18.5)
Dislocation	7 (6.5)	20 (27.7)
Fracture	6 (5.6.)	4 (4.9)
Other (persistent pain, impingement, implant malposition)	10 (9.3)	6 (7.4)

Of the revised THAs, 33 (38.8%) in the MoM group and 38 (48.1%) in the CoP group had a total revision, while 44 (51.8%) THAs in the MoM group and 24 (30.4%) THAs in the CoP group had only a cup revision with or without head exchange, and two (2.4%) MoM THAs and nine (11.4%) CoP THAs had a stem revision only. The remaining six (7.1) MoM and 8 (10.1) CoP THAs revised had other types of surgical procedures, such as head exchange only or prosthesis removal (CoP THA). In two (2.4%) cases (MoM THA), the type of revision surgery was unknown because performed in another hospital.

In the MoM group, 30 THAs had a reoperation at a median time of 48 months (IQR 10.8-129.8) and 66 THAs in the CoP group at a median time of 22.5 months (IQR 4.8–67.3).

Prosthetic joint infection occurred in 17 (2%) MoM THAs at a median time of 52.5 months (IQR 10.7-135.8); and in 21 (0.9%) CoP THAs at a median time of 35.9 months (IQR 3.7–76.7). The adjusted HR for prosthetic joint infection was 2.1 (95% CI 1.0 to 4.1). Median time for dislocation was 5.0 months (IQR 0.5–156) for MoM THAs and 0.8 months (IQR 0.4–3.2) for CoP THAs. Adjusted HR for dislocation was 1.0 (95% CI 0.6 to 1.6). Periprosthetic fracture occurred at a median time of 45.4 months (IQR 3.9–45.4) in the CoP group and 113.8 months (IQR 57.9-148.3) in the MoM group. Adjusted HR for periprosthetic fracture was 1.0 (95% CI 0.5 to 2.1) (Table 2).

**Fig. 1:**
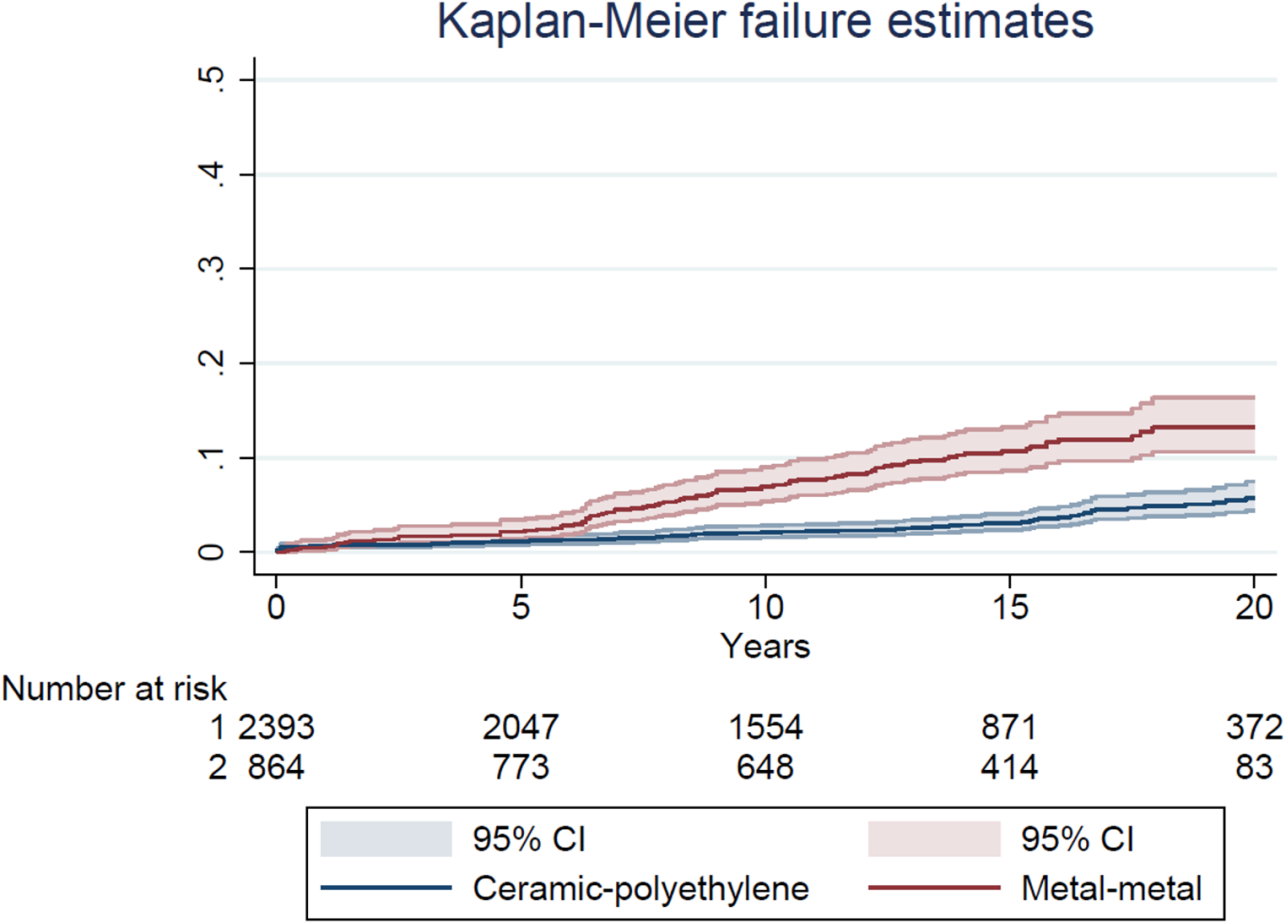
Kaplan-Meier failure estimates with endpoint all-cause revision

**Fig. 2:**
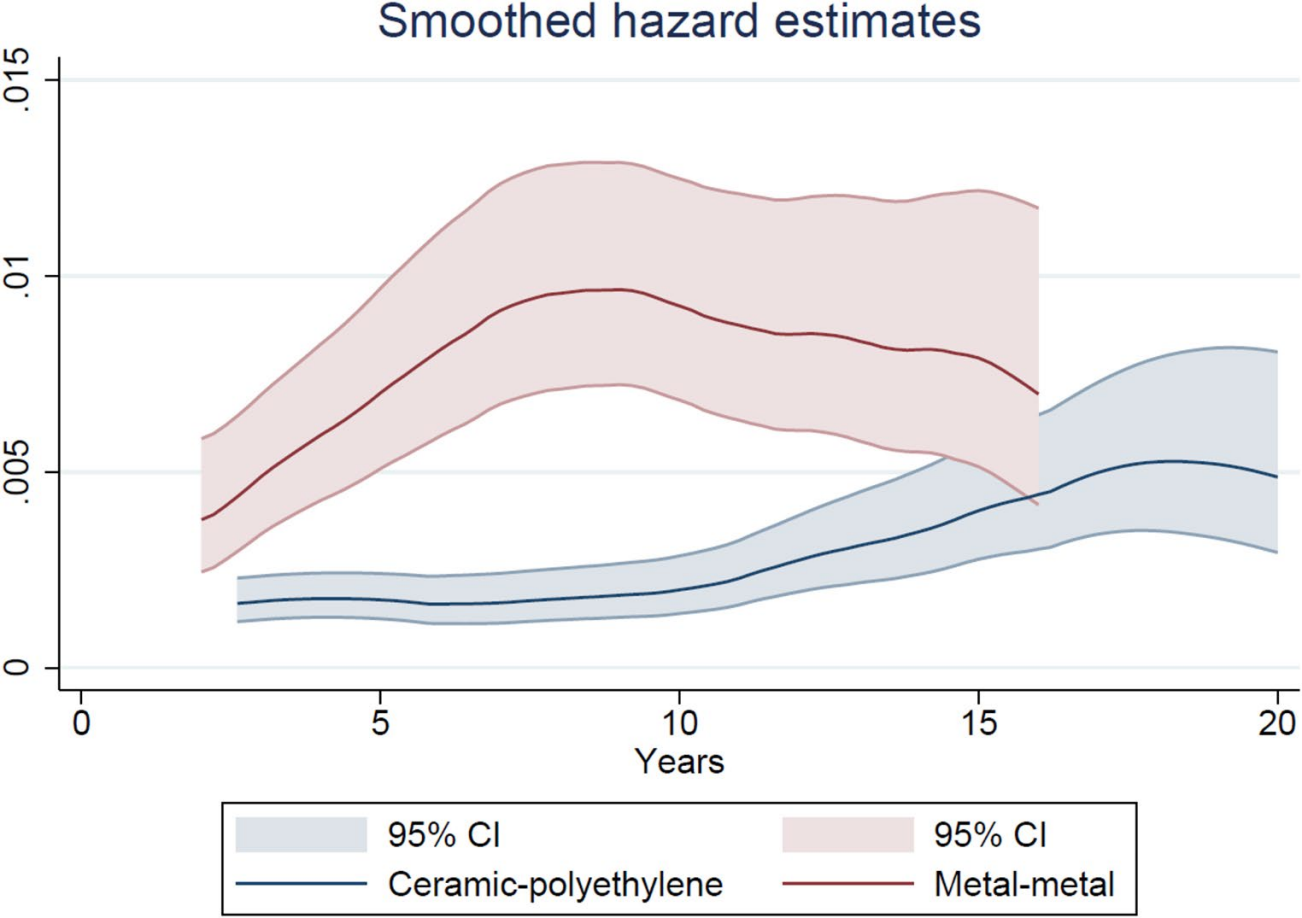
Smoothed hazard estimates

Cumulative risk of all-cause revision (CRR) at 20 years was 13.2% (95% CI 10.6 to16.3) in the MoM group and 6.3% (95% CI 4.8 to 8.3) in the CoP group (log rank test *p* < 0.001) (Fig. 1). We observed an increasing revision rate for the MoM THAs over time, at five years the revision rate was 2.8% (95% CI 1.9 to 4.2), 7.7% (95% CI 6.0 to 9.9) at ten years, and 11.7 (95% CI 9.4 to 14.4) at 15 years. The smoothed hazard function revealed the largest difference in instantaneous revision rate between three and 14 years postoperative. After that period no difference was observed (Fig. 2).

During the inclusion period, 300 (34.7%) deaths (any cause) occurred in the MoM group and 1690 (70.6%) in the CoP group. Unadjusted and adjusted HRs for all-cause mortality, the latter taking into account sex, age at surgery, diagnosis, type of stem fixation, comorbidity count, BMI, ASA score and smoking status, were 0.44 (95% CI 0.39 to 0.50) and 0.83 (95% CI 0.72 to 0.94).

## Discussion

This study compared long-term outcomes of small head MoM and CoP THAs all with the same cup. The results showed a higher all-cause revision rate in the MoM compared to the CoP group. Twenty years after surgery the cumulative risk of all-cause revision was twice as high in patients with a small head MoM as compared to a CoP THA. Most of the excess in revisions among MoM patients occurred between three and 14 years postoperative. Incidence of prosthetic joint infection was more than twice as high in the MoM group compared to the CoP group. However, outcomes dislocation and periprosthetic fracture were similar. Finally, rate of mortality was less in the MoM group before adjustment and remained slightly less thereafter.

Previous studies with end point revision for any reason showed similar survivals at mid- and long-term follow up, ranging from 91 to 94% at 13 and 14 years, and 87–92.2% after 16 to 20 years, but unlike our study included only small number of implants (THAs included 52 to 180) [10–13, 15, 27]. Data from the Australian Arthroplasty Registry [28] reported revision rates for small head Metasul THAs comparable to our results, and also increasing over time, almost 7% at ten years, more than 9% at 15 years, and 12% by 20 years. Just as we observed that from three years the difference in the instantaneous revision rate increased, another study also observed a decreased in survival at four years [29], corresponding to the moment the monitoring of patients with MoM THAs with head ≤ 36 mm changed and became more thorough. That we observed a higher revision rate in women than in men with MoM bearing compared to CoP is supported by what was published previously. Data from the National Joint Registry of England and Wales [30] showed a higher revision rate for 28 mm MoM THA in women than in men. This sex difference was also observed in hip resurfacing arthroplasty [31], and could be explained by a higher rate and severity of metal sensitization in women [32].

Published case-series already reported ALTR in revised small head MoM THAs, but with a low prevalence (less than 2%) [3–8]. In our study more than one third of the revised MoM THA had ALTR diagnosed. Data from the Australian Arthroplasty Registry showed increased revision for ALTR in small head MoM THA over time compared to other bearings [33]. Problems that occurred with MoM prostheses changed the monitoring of patients with MoM THAs, with the introduction of imaging studies in symptomatic patients or for patients with elevated blood ion levels, consequently increasing the diagnosis of ALTR and the indication for revision. The change in follow up of MoM THAs patients might explain the increase in ALTR diagnosed over time.

The incidence rate for infection was higher in the MoM group, and occurred earlier on a shorter time interval compared to the CoP group. Previous studies already assessed the infection rate in MoM THAs and observed an elevated rate of infection compared to other bearings [34, 35], just as our results showed. However, these studies included only large head MoM THAs. Data from the British Arthroplasty Registry also showed higher rate of revision for infection for MoM THAs compared to CoP THAs, 1.21 (8.87 to 1.69) vs. 0.92 (0.85 to 1.01) per 1’000 pers-years [36]. But they did not stratify between large and small head size. Metal debris may predispose to infection by acting on the immune system and on bacterial growth [37], but so far little data is available on infection in small head MoM THAs and the potential predisposition of metal debris to infection. In our study, we did not look at the metal ions levels in the MoM group, so it is difficult to draw conclusions about the influence of metal debris on the infection rate in the MoM group.

As expected, our MoM patient cohort was younger than the CoP cohort. Indeed, because it was thought that the mechanical properties of the 2nd generation MoM prostheses would be better than the bearings with polyethylene [38], THA with MoM bearings were mainly implanted in younger patients. Although the revision rate was higher in the MoM group, we did not observe a higher mortality rate in the MoM group, the mortality rate remained slightly less even after adjustment. In a recent large cohort study, patients with a primary THA containing cobalt-chromium had no increase in all-cause mortality compared to patients with non-cobalt-chromium containing primary THA [39]. These results are reassuring, knowing that a high number of patients worldwide still have THAs with small head MoM bearing.

Our study has some limitations. First, because of the observational nature of the study, confounding by indication cannot be excluded when comparing the two types of bearings. To minimize this, adjustment was performed for baseline imbalances. Missingness of covariate information was very low (0–1% for all potential confounders except for smoking status where it was 9%). Second, the information on presence of ALTR was only reported for revised MoM THAs, however, the occurrence of ALTR is likely higher due to its presence in asymptomatic patients [40]. Third, the slightly lower mortality rate in MoM patients might be due to residual confounding.

## Conclusion

Our results showed two-times higher cumulative risks of all-cause revision and of prosthetic joint infection in patients with a small head MoM as compared to those with a CoP THA over a follow-up period of 20 years. However, most of the excess in revisions among MoM patients occurred between 3 and 14 years postoperative, and after that period no difference was observed.

## Data Availability

No datasets were generated or analysed during the current study.
